# Pilot trial of high-dose vitamin C in critically ill COVID-19 patients

**DOI:** 10.1186/s13613-020-00792-3

**Published:** 2021-01-09

**Authors:** Jing Zhang, Xin Rao, Yiming Li, Yuan Zhu, Fang Liu, Guangling Guo, Guoshi Luo, Zhongji Meng, Daniel De Backer, Hui Xiang, Zhiyong Peng

**Affiliations:** 1 Dept. of Critical Care Medicine, Zhongnan Hospital of Wuhan University, Wuhan, 430071 Hubei China; 2 Anti-Aging Medical Center, Taihe Hospital, Huibei University of Medicine, Shiyan, 442000 Hubei China; 3 Department of Pulmonary and Critical Care Medicine, Taihe Hospital, Huibei University of Medicine, Shiyan, 442000 Hubei China; 4 Department of Infectious Diseases, Taihe Hospital, Huibei University of Medicine, Shiyan, 442000 Hubei China; 5 Department of Intensive Care, CHIREC Hospitals, Université Libre de Bruxelles, Brussels, Belgium; 6 Clinical Research Center of Hubei Critical Care Medicine, Wuhan, 430071 Hubei China

**Keywords:** High-dose intravenous vitamin C, Coronavirus disease 2019, Severe acute respiratory syndrome coronavirus 2

## Abstract

**Background:**

Few specific medications have been proven effective for the treatment of patients with severe coronavirus disease 2019 (COVID-19). Here, we tested whether high-dose vitamin C infusion was effective for severe COVID-19.

**Methods:**

This randomized, controlled, clinical trial was performed at 3 hospitals in Hubei, China. Patients with confirmed severe acute respiratory syndrome coronavirus 2 (SARS-CoV-2) infection in the ICU were randomly assigned in as 1:1 ratio to either the high-dose intravenous vitamin C (HDIVC) or the placebo. HDIVC group received 12 g of vitamin C/50 ml every 12 h for 7 days at a rate of 12 ml/hour, and the placebo group received bacteriostatic water for injection in the same way within 48 h of arrival to ICU. The primary outcome was invasive mechanical ventilation-free days in 28 days (IMVFD28). Secondary outcomes were 28-day mortality, organ failure (Sequential Organ Failure Assessment (SOFA) score), and inflammation progression (interleukin-6).

**Results:**

Only 56 critical COVID-19 patients were ultimately recruited due to the early control of the outbreak. There was no difference in IMVFD28 between two groups (26.0 [9.0–28.0] in HDIVC vs 22.0 [8.50–28.0] in control, *p* = 0.57). HDIVC failed to reduce 28-day mortality (*P* = 0.27). During the 7-day treatment period, patients in the HDIVC group had a steady rise in the PaO_2_/FiO_2_ (day 7: 229 vs. 151 mmHg, 95% CI 33 to 122, *P* = 0.01), which was not observed in the control group. IL-6 in the HDIVC group was lower than that in the control group (19.42 vs. 158.00; 95% CI -301.72 to -29.79; *P* = 0.04) on day 7.

**Conclusion:**

This pilot trial showed that HDIVC failed to improve IMVFD28, but might show a potential signal of benefit in oxygenation for critically ill patients with COVID-19 improving PaO2/FiO2 even though.

## Introduction

Severe acute respiratory syndrome coronavirus 2 (SARS-CoV-2) infection has become a global health issue [1, 2]. While the majority of patients presented with mild symptoms and did not even need hospitalization [3], nearly 30% of adult patients suffer from severe pneumonia and acute respiratory distress syndrome (ARDS), often associated with sepsis or septic shock, and multiple organ (kidney, liver, and heart) failure. Patients with ARDS and systemic complications require critical care and lead to a higher risk of death [4, 5, 6]. Due to the lack of effective medications against SARS-COV-2, the main management is supportive therapy.

Similar to the pathophysiology of severe acute respiratory syndrome (SARS)-related ARDS, SARS-CoV-2 infection stimulates the innate immune system, causing numerous types of cytokine release, namely, a “cytokine storm”, inducing systemic inflammatory response [7, 8] and multiple organ failure [9, 10]. A retrospective study on SARS suggested that the worsening after 2 weeks was not related to uncontrolled viral replication, but related to immunopathological damage [11]. Therefore, antiviral therapy alone may be insufficient to treat COVID-19 patients.

Vitamin C (ascorbic acid, ascorbate) functions as a potent water-soluble antioxidant by directly scavenging oxygen free radicals and acting as an essential co-factor for the production of catecholamines, vasopressin, and cortisol in the human body [12]. Vitamin C is also found in high concentrations in leukocytes and implicated in several immune responses and functions [13]. Emerging evidence in preclinical studies indicated that vitamin C played a crucial role in ameliorating the effects of inflammation by inhibiting proinflammatory cytokine production, assisting immunoregulation, neutralizing reactive oxygen species (ROS), and protecting host cells [14, 15]. Hypovitaminosis C was ubiquitous in critically ill patients, and approximately 40% of the patients had a severe deficiency [16], while the low vitamin C serum level cannot be corrected by oral supplementation due to the issue of pharmacokinetics [17]. In a latest research, of 18 adult ICU patients COVID-19 who met ARDS criteria, 94.4% had undetectable vitamin C levels and 1 patient had low levels [18]. Thus, high-dose intravenous vitamin C (HDIVC) was added to the standard therapy of critically ill patients in recent studies, such as sepsis [19–21], ARDS [21, 22], cardiac surgery [23], and burn [24]. The results showed that HDIVC was safe for critically ill patients and significantly reduced vasopressor support [25], limited organ injury [26], shortened the duration of mechanical ventilation [27] and ICU stay [28], and safety/feasibility in severe sepsis [19]. Additionally, vitamin C has direct nonspecific antiviral activity in vitro [29], although it is unclear whether this confers any protection to humans with COVID-19.

Therefore, we hypothesized that HDIVC together with conventional treatments would improve the outcomes for adult patients admitted to the ICU due to severe COVID-19 by preventing cytokine storms and reducing lung and other organ injuries. In this context, we conducted this multicenter, randomized, blind clinical trial to provide a therapeutic strategy for critically ill patients with COVID-19.

## Methods

This study is a multicenter, randomized trial that was approved by the ethics committee of Zhongnan Hospital of Wuhan University (#2020001). This study was conducted in the ICUs of Zhongnan Hospital of Wuhan University, Leishenshan (Thunder God Mountain) Hospital, and Taihe Hospital from February 14, 2020, to March 29, 2020. The ICUs specifically for COVID-19 from Zhongnan Hospital and Leishenshan Hospital were managed by the same team. The trial was registered on the website of ClinicalTrials.gov (ID: NCT04264533; registered February 14 2020) before patient recruitment.

## Patient enrollment

Patients were screened and enrolled following admission to the three ICUs. The patients who were diagnosed as severe SARS-CoV-2-related pneumonia, appeared or had a high risk of multiple organs injury would be transferred to ICU. The following inclusion criteria were met: (1) age  ≥ 18 and < 80 years; (2) RT-PCR positive for SARS-CoV-2; (3) pneumonia confirmed by chest imaging and admission to the ICU; (3) PaO_2_/FiO_2_(P/F) < 300 mmHg. Exclusion criteria were allergy to vitamin C, pregnancy or breastfeeding, expected survival duration < 24 h, and previous history of glucose-6-phosphate dehydrogenase deficiency or end-stage pulmonary disease. Patients who were already enrolled in other clinical trials were excluded as well. If these criteria were met within 48 h of ICU admission, informed consent was obtained from the patients or their family members. The reason was because the efficacies of the treatments could not be evaluated with limited times of treatment.

## Randomization, allocation and blinding

Each ICU was assigned with an independent random numeric table generated by Microsoft Excel 2019 by the primary investigator alone. Each table had equal numbers of 1 and 2, which represented the placebo group (bacteriostatic water infusion) and treatment group (HDIVC), respectively. The generated random list was stored by the principal investigator who was not involved in the treatment of patients and hidden to the other investigators. When a patient was transferred to the ICU and met the enrollment criteria, the clinician on duty would inform the principal investigator and obtain a number from the list. Then, participants were enrolled in the corresponding group according to the chronological order of ICU recruitment. The grouping and intervention were unknown to the participants and investigators who were responsible for data collection and statistical analysis. VC injection and sterile water for injection were both colorless and contained in the same brown syringes with different marks and without explanations on the syringe to make sure that patients could not distinguish the treatment they receive.

## Study interventions

Patients were randomized to receive vitamin C or placebo within 48 h after admission to the ICU. To control the infusion rates accurately and not affect the fluid management of severe patients, we infused vitamin C or placebo via central vein catheterization controlled by a pump. The study groups in this trial were (1) HDIVC: 24 g vitamin C per day. Patients were infused with 12 g vitamin C diluted in 50 ml of bacteriostatic water every 12 h at a rate of 12 ml/hour by infusion pump for 7 days. (2) Placebo: 50 ml of bacteriostatic water infused every 12 h at the same rate. Study interventions were initiated on the same day as informed consent and randomization. The preparation, transportation, storage, and use of therapies (VC and bacteriostatic water for injection) were in line with the drug management protocol in each hospital.

### General treatments and standard procedure of ventilation supports

In addition, other general treatments followed the latest COVID-19 guidelines [30]. Oseltamivir and azithromycin were usually used in the general ward. After ICU admission, low weight molecular heparin was applied for the prevention deep vein thrombus. Piperacillin/tazobactam was used for patients receiving tracheal intubation.

If the patients showed the symptoms of rapid deterioration of hypoxemia, severe ARDS, or septic shock, hydrocortisone (1 mg/kg/day) could be considered.

Respiratory support (IMV, NIV and HFNC) were given to patients with hypoxic respiratory failure and ARDS. If respiratory failure could not be improved or worsened continuously within a short time after using HFNC or NIV, intubation were performed and the approach of lung-protective ventilation was applied. ECMO was considered as the rescue therapy when the refractory hypoxemia was difficult to be corrected by protective lung ventilation [4]. When patients’ respiratory functions improved and were ready for weaning from the ventilators, the spontaneous breathing test (SBT) was performed. After the SBT was passed, invasive ventilator was considered to remove with the endotracheal tube extubation.

### Risks and adverse events

Adverse events (AEs) related to HDIVC included (1) nausea or vomiting during or after infusion of VC; (2) electrolyte disturbance; and (3) acute kidney injury, as described by Khoshnam-Rad [31]. AEs and serious adverse events (SAEs) were observed and followed in accordance with the good clinical practice guidelines issued by the National Medical Products Administration of the People’s Republic of China. If any severe adverse events were observed during infusion, the infusion was stopped immediately, and the patient’s vital signs were carefully monitored. All the AEs and SAEs were recorded in detail, and the causal relationship between the infusion and AEs was analyzed.

## Data collection and management

Baseline data, which included demographics, anthropometrics, comorbid conditions, vital signs, Acute Physiology and Chronic Health Evaluation II (APACHE II) scores, and Glasgow Coma Scale (GCS) scores, were obtained on the day of randomization. Laboratory data, Sequential Organ Failure Assessment (SOFA) scores, PaO_2_/FiO_2_, and other treatments used were monitored on days 1, 3, and 7 (day 1 was defined as the day of the first administration of study drug).

The primary outcome of the study was invasive mechanical ventilation (IMV)-free days in 28 days (IMVFD28). Secondary outcomes included 28-day mortality, organ functions and inflammatory parameters, including white blood cell counts, neutrophil counts, lymphocyte counts, procalcitonin, interleukin-6 (IL-6), and C-reactive protein (CRP). Multi-organ dysfunction was assessed using SOFA scores. Additionally, vasopressor days, respiratory support days (including invasive and noninvasive mechanical ventilation), IMVFD28, patient condition improvement rate, patient condition deterioration rate, length of ICU and hospital stay, ICU and in-hospital mortality were recorded as additional secondary outcomes of this research. IMVFD28s were defined as the number of days a patient was extubated after recruitment to day 28. If the patient died with MV, a value of zero was assigned. Deterioration of the patient’s condition was defined as the patient requiring HFNC or NIV on day 1 and requiring ECMO or IMV, or dying, after 7 days of treatment. Improvement of the patient’s condition was defined as the patient requiring ECMO or IMV on day 1 and switching to HFNC, NIV, or discharged from the ICU after 7 days of treatment. The P/F was calculated based on the PaO_2_/FiO_2_, and we choose the lowest values recorded on the specific day. All the data were collected from the clinical information system of three ICUs. Septic shock was identified according to International Guidelines for Management of Sepsis and Septic Shock (2016). Acute kidney injury was identified according to the Kidney Disease: Improving Global Outcomes definition. Acute cardiac injury was defined as the serum levels of troponin I were above the 99th percentile upper reference limit or new abnormalities were shown in electrocardiography and echocardiography. Acute liver failure (ALF), which is defined as coagulopathy (INR ≥ 1.5), hepatic encephalopathy, and onset less than 26 weeks in a patient without underlying chronic liver disease. Coagulation disorders were defined as the presence of D-dimer > 0.24 mg/L or FDP > 5 mg/L.

## Statistical analysis

The sample size was calculated according to primary endpoint, as this trial began at the early stage of COVID-19, such preliminary data lacked, and the sample size was finally calculated from the previous studies on ARDS [21]. We used the non-inferiority test formula to calculate the sample size with a one-sided error rate (α) of 2.5%, a power of 80%, and a withdrawal rate of 10%, and the anticipated sample size was 140. With the control of the epidemic, this trial was stopped early, and the number of qualifying COVID-19 patients did not satisfy the anticipated sample size. Thus, we considered this trial as the pilot trial. Numerical variables are described as the mean with standard deviation (SD) or median with interquartile range (IQR) according to distribution and were compared with the t-test/Mann–Whitney U test. Category data are represented as frequencies and proportions and compared with the Chi-square test and Fisher's exact test. The primary intention-to-treat analysis included all randomized participants. For the outcome variables, the hazard ratio and 95% CI were estimated by the Cox proportional risk model for mortality, and odds ratios with 95% CI were calculated by binary logistic regression for the other variables. Kaplan–Meier analysis was used to estimate the 28-day mortality to reflect the early survival differences for the two groups, and survival curves were compared with the Wilcoxon test. Survival analyses were further performed in subgroup with SOFA score more than 2. The testing was 2-sided, and a *P*-value < 0.05 was considered statistically significant. SPSS 20.0 and GraphPad Prism 8.0 were used to complete data processing and statistical analysis.

## Results

### Baseline characteristics of the patients

**Fig. 1:**
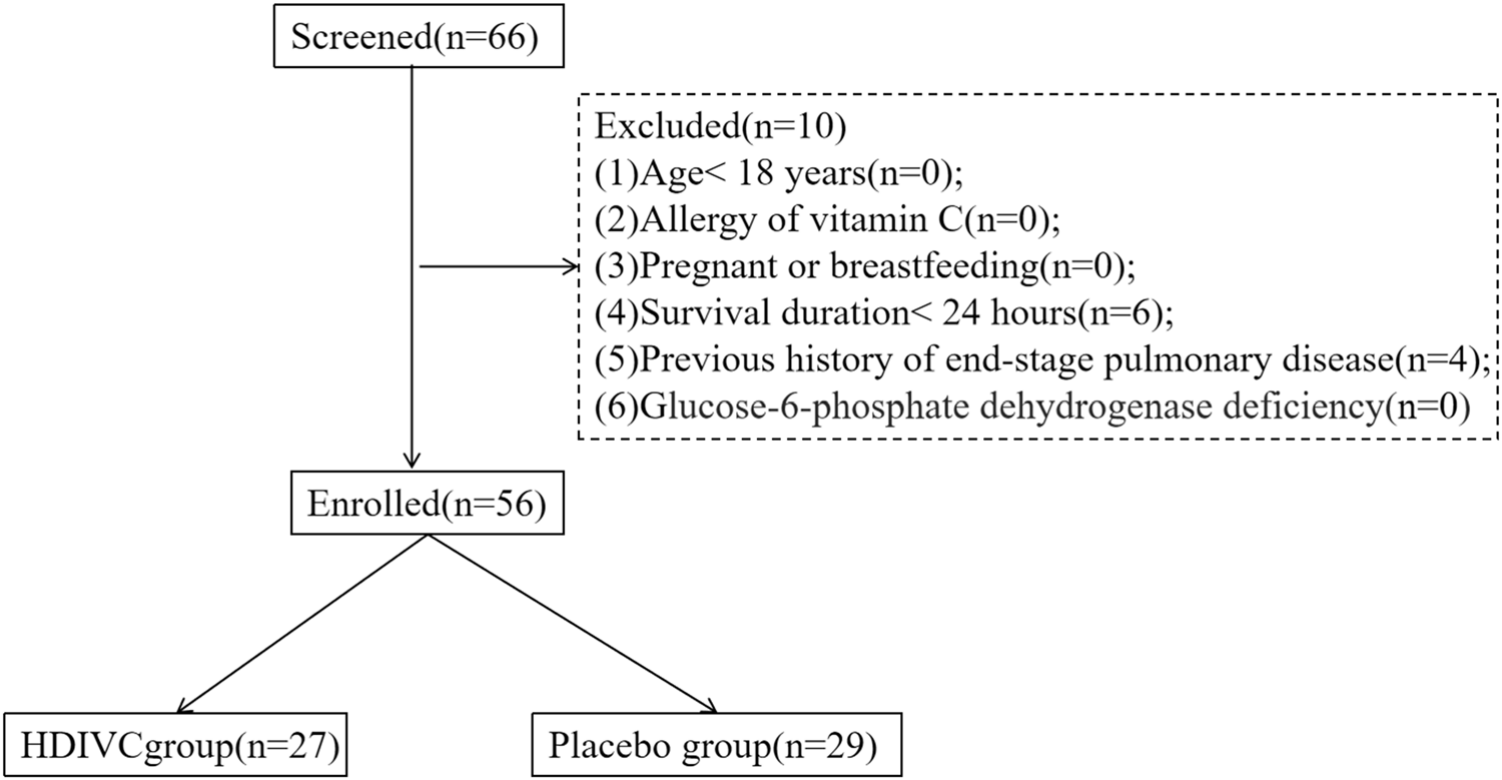
Flowchart of patients. *HDIVC* high-dose intravenous vitamin C

A total of 66 patients were identified (Fig. 1), 56 patients of them were enrolled and randomized in this study from February 14, 2020, to March 29, 2020. Patients were enrolled in the Leishenshan (Thunder God Mountain) Hospital (39 patients), Zhongnan Hospital of Wuhan University (11 patients), and Taihe Hospital of Hubei University of Medicine (6 patients). All participants (56) were included in the primary intention-to-treat analysis, 50 (89.2%) received the full 7-day treatment course, 4 (7.14%) only received 5 or 6 days of treatment due to discharge from the ICU, and 2 of them only received treatment for less than 3 days due to early death of natural process. Tables 1 and 2 shows the baseline demographic and clinical characteristics of the 56 patients.

**Table 1:** Baseline characteristics of intention-to-treat patients

Variable	All patients (*n* = 56)	Vitamin C (*n* = 27)	Placebo (n = 29)	*P* Value
Demographics				
Age, years	66.7 ± 12.7	66.3 ± 11.2	67.0 ± 14.3	0.86
Gender, male, n, %	36(66.1)	15(55.6)	22(75.9)	0.09
Height, cm	168.8 ± 6.6	167.0 ± 6.9	170.8 ± 5.8	0.08
Weight, kg	62.0 ± 10.5	59.7 ± 11.2	64.4 ± 9.4	0.16
Centers				
Zhongnan Hospital of Wuhan University, *n*, %	11(19.6)	5(18.5)	6(20.6)	-
Leishenshan (Thunder God Mountain) Hospital, *n*, %	39(69.6)	19(70.4)	20(69.0)	-
Taihe Hospital, *n*, %	6(10.7)	3(11.1)	3(10.3)	-
General condition on randomization day				
Highest temperature,℃	37.4 ± 1.0	37.3 ± 0.8	37.4 ± 1.1	0.65
Highest heart rate, times/min	92.4 ± 18.5	95.3 ± 19.2	89.8 ± 17.8	0.27
Lowest MAP, mmHg	91.0 ± 17.9	88.4 ± 16.6	93.4 ± 18.9	0.49
Highest RR, times/min	25[20–36]	25[21–31]	24[20–30]	0.19
Lowest SPO_2_, %	93[88–98]	93[81–98]	93[90–97]	0.93
APACHE II score	13.5[10.3–15.8]	14.0[11.0–16.0]	13.0[9.5–15.0]	0.24
GCS score	15.0[14.5–15.0]	15.0[13.0–15.0]	15.0[15.0–15.0]	0.75
Comorbidities, n, %				
Diabetes	17(30.4)	8(29.6)	9(32.1)	0.57
Hypertension	25(44.6)	10(37.0)	15(51.7)	0.20
Coronary heart disease	12(21.4)	4(14.80)	8(27.6)	0.33
Chronic lung disease	3(5.4)	1(3.7)	2(6.9)	1.00
Chronic renal failure	1(1.8)	1(3.7)	0(0.0)	0.48
Malignant tumor	3(5.4)	3(11.1)	0(0.0)	0.11
Nervous system diseases	11(20.4)	7(25.9)	4(13.8)	0.32
Median duration of symptoms before HDIVC therapy, days	17.0[11.0–25.0]	22.0[11.0–33.0]	15.0[11.0–22.0]	0.18
Other treatments during 7 days HDIVC therapy				
Corticosteroid use, *n*, %	18(32.1)	8(36.4)	10(38.5)	1.00
Antibiotic, *n*, %	51(91.1)	24(92.3)	27 (96.4)	1.00
Net fluid balance, mL/24 h				
Day 1	190[-1487–662]	252[-252–810]	155[-520–499]	0.39
Day 2	156[-349 -653]	192[-508–883]	121[-90 -577]	0.94
Day 3	62[-703–768]	-240[-1004 -233]	463[5–1351]	0.02

Data were expressed as mean ± standard deviation, as median [interquartile range], or as numbers (percentage). Comparisons were performed using Student’s t test, Wilcoxon–Man–Whitney, Chi-square, or Fisher’s exact

*SD* standard deviation, *IQR* interquartile range, *APACHE* Acute Physiology and Chronic Health Evaluation, *GCS* Glasgow Coma Scale, *HDIVC* high-dose intravenous vitamin C

**Table 2:** Outcomes in a trial of HDIVC in patients with COVID-19

Variable	Day	Vitamin C (*n* = 27)	Placebo (*n* = 29)	Difference, coefficient (95% CI)	P value
IMVFD28, days		26.0[9.0–28.0]	22.0[8.5–28.0]	1.3(− 4.7 to 7.2)	0.57
IMV days to day 28, days		1.5[0.0-19.0]	6.0[0.0–16.0]	− 0.8(− 6.4 to 4.9)	0.60
HFNC days to day 28, days		0.5[0.0-8.3]	2.0[0.0 -7.0]	0.2(− 2.9 to 3.3)	0.85
NIV days to day 28, days		0.0[0.0 -3.3]	0.0[0.0-1.8]	1.2(− 1.2 to 3.7)	0.68
Patients’ condition deterioration, n, %		3(11.5)	6(24.0)	0.4(0.1 to 1.7)	0.19
Patients’ condition improvement, n, %		5(19.2)	6(21.4)	0.9(0.2 to 3.3)	0.84
ICU mortality, n, %		6(22.2)	11(37.9)	HR 0.5(0.2 to 1.5)	0.20
ICU mortality of patients with SOFA ≥ 3, n, %		5(21.7)	11(52.4)	HR 0.2(0.1 to 0.9)	0.04
ICU stay, days		22.9 ± 14.8	17.8 ± 13.3	5.0(− 2.5 to 12.7)	0.20
Hospital mortality, n, %		6(22.2)	11(37.9)	HR 0.5(0.2 to 1.5)	0.20
Hospital mortality of patients with SOFA ≥ 3, n, %		5(21.7)	11(52.4)	HR 0.2(0.1 to 0.9)	0.04
Hospital stay, days		35.0 ± 17.0	32.8 ± 17.0	2.2(− 7.5 to 11.8)	0.65
28-day mortality, n, %		6(22.2)	10(34.5)	HR 0.5(0.2 to 1.8)	0.31
28 days mortality of patients with SOFA ≥ 3, n, %		5(21.7)	10(47.6)	HR 0.3(0.1 to 1.1)	0.07
SOFA scores					
	1	3.5[3–6.8]	2.0[3.0–5.0]	0.7(− 0.9 to 2.3)	0.37
	3	4.0[2.0–8.0]	4.0[3.0–7.0]	− 0.3(− 2.6 to 1.9)	0.50
	7	3.0[2.0–5.8]	6.0[2.50–8.0]	− 1.14(− 3.1 to 0.8)	0.24
	△7	0.0[-2.75–1.0]	0.0[–1.0–3.5]	− 1.35(-3.04− 0.34)	0.25
Lowest P/F					
	1	188.7 ± 95.4	210.6 ± 128.5	34.6(− 91.9 to 48.0)	0.53
	3	217.3 ± 96.5	189.5 ± 101.9	30.7(− 34.3 to 89.9)	0.37
	7	228.5 ± 72.6	150.7 ± 75.3	22.1(33.2 to 122.5)	0.01
	△7	20.0 ± 96.68	− 51.88 ± 150.72	41.02(5.92-172.45)	0.04
Lowest MAP					
	1	88.4 ± 16.6	93.4 ± 18.9	− 3.34(− 13.08 -6.38)	0.49
	3	87.6 ± 12.42	91.00 ± 14.00	− 3.40(− 10.74 -3.94)	0.36
	7	87.74 ± 14.24	88.77 ± 10.97	− 1.03(− 8.58 -6.53)	0.79
Advanced life support, n, %					
CRRT					
	1	1(3.8)	3(10.7)	OR 0.3(0.0 to 3.5)	0.61
	7	3(12.5)	1(3.8)	OR 3.57(0.4 to 36.9)	0.34
ECMO					
	1	1(3.8)	2(7.1)	OR 0.5(0.0 to 6.0)	1.00
	7	0(0.0)	2(9.1)	OR 0.5(0.4 to 0.7)	0.50
Oxygen-support category					
HFNC					
	1	7(25.9)	11(37.9)	OR 0.6(0.2 to 1.8)	0.40
	7	11(47.8)	9(39.1)	OR 14.3(0.4 to 4.6)	0.77
NIV					
	1	7(25.9)	7(24.1)	OR 1.1(0.3 to 3.7)	1.00
	7	7(30.4)	2(8.7)	OR 4.6(0.8 to 25.2)	0.14
IMV					
	1	11(40.7)	12(41.3)	OR 1.0(0.3 to 2.9)	1.00
	7	10(43.5)	11(47.8)	OR 0.8(0.3 to 2.7)	1.00
Complications, n, %					
Septic shock	9(34.6)	8(28.6)	OR 1.3(0.4 to 2.4)	0.77	
Acute cardiac injury	7(26.9)	13(48.1)	OR 0.4(0.1 to 1.3)	0.16	
Acute liver injury	12(48.0)	13(48.1)	OR 1.0(0.3 to 3.0)	1.00	
Acute kidney injury	3(12.0)	6(22.2)	OR 0.5(0.1 to 2.2)	0.50	
Coagulation disorders	9(34.6)	7(25.9)	OR 1.5(0.5 to 5.0)	0.56	

Data were expressed as mean ± standard deviation, as median [interquartile range], or as numbers (percentage). Hazard ratio and 95% CI were estimated by Cox proportional risk model. Odds ratio with 95% CI were calculated by binary logistic regression for the rest. Absolute difference was expressed as a percentage with the 95% CI range. P values were calculated by logistic regression. △7 means the difference between the value from Day 1 to Day 7

*IMVFD28* invasive mechanical ventilation-free days in 28 days, *HDIVC * high-dose intravenous vitamin C, *COVID-19* coronavirus disease 2019, *SD* standard deviation; IQR, interquartile range; *HR* hazard ratio, *OR* odds ratio, *CI* confidence interval, *SOFA* Sequential Organ Failure Assessment, *P/F* PaO_2_/FiO_2_, *MAP* mean arterial pressure; CRRT, continuous renal replacement therapy, *ECMO* extracorporeal membrane oxygenation, *HFNC* high flow nasal cannula, *IV* invasive ventilation; IMV, invasive mechanical ventilation, *NIV* noninvasive mechanical ventilation, *ICU* intensive care unit

The average age of the study patients was 66.7 ± 12.7 years, and 66.1% of the patients were male. The APACHE II score of all patients was 13.5 (IQR, 10.2-15.7), with no differences between groups. The most common comorbidity was hypertension (44%), followed by diabetes (30%) and coronary heart disease (22%). The average time from symptom onset to starting HDIVC treatment was 17 (11–25) days. No significant differences in vital signs, laboratory results, disease severity, or treatments were observed between groups at baseline.

### Primary outcome

**Fig. 2:**
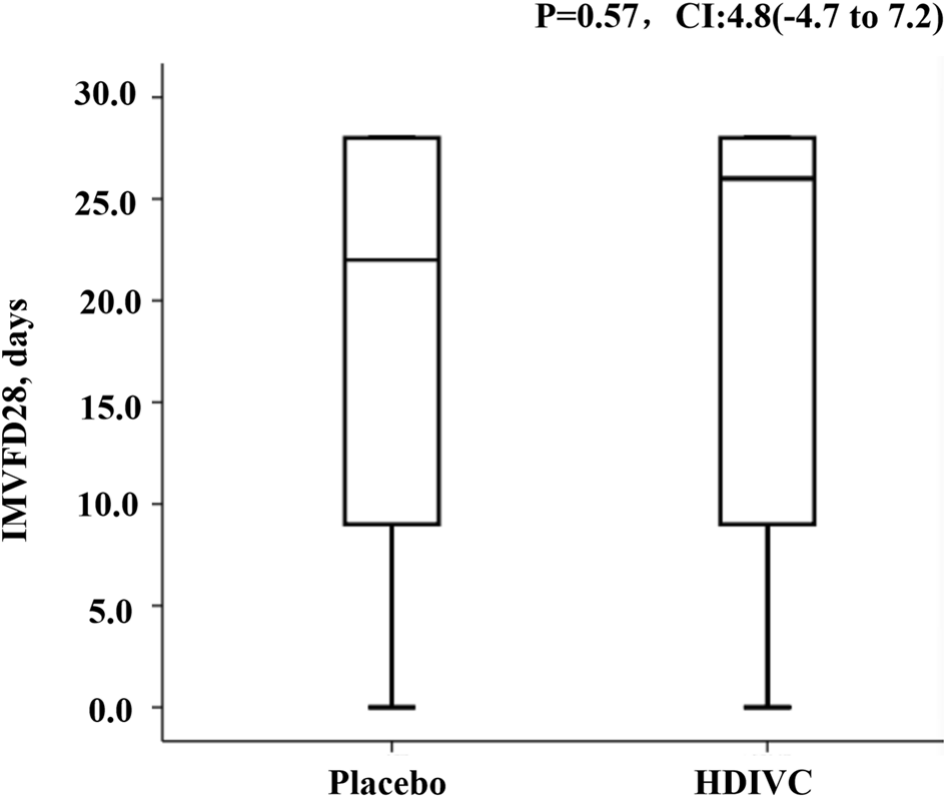
The IMVFD28 in high-dose intravenous vitamin C and placebo group. The IMVFD28 was 26.0 days[9.0–28.0] in HDIVC, and 22.0 days[8.5–28.0] in placebo group, but this difference was not statistically significant (*P* = 0.57, CI 4.8[-4.7 to 7.2]). *IMV* invasive mechanical ventilation, *HDIVC* high-dose intravenous vitamin C

The IMVFD28 was 26.0 days [9.0–28.0] in HDIVC, and 22.0 days [8.50–28.0] in placebo group, but this difference was not statistically significant (P = 0.57, HR, CI: 4.8[-4.7 to 7.2]) (Fig. 2). The post hoc computation of power for IMVFD28 was 0.3.

### Secondary outcomes

**Fig. 3:**
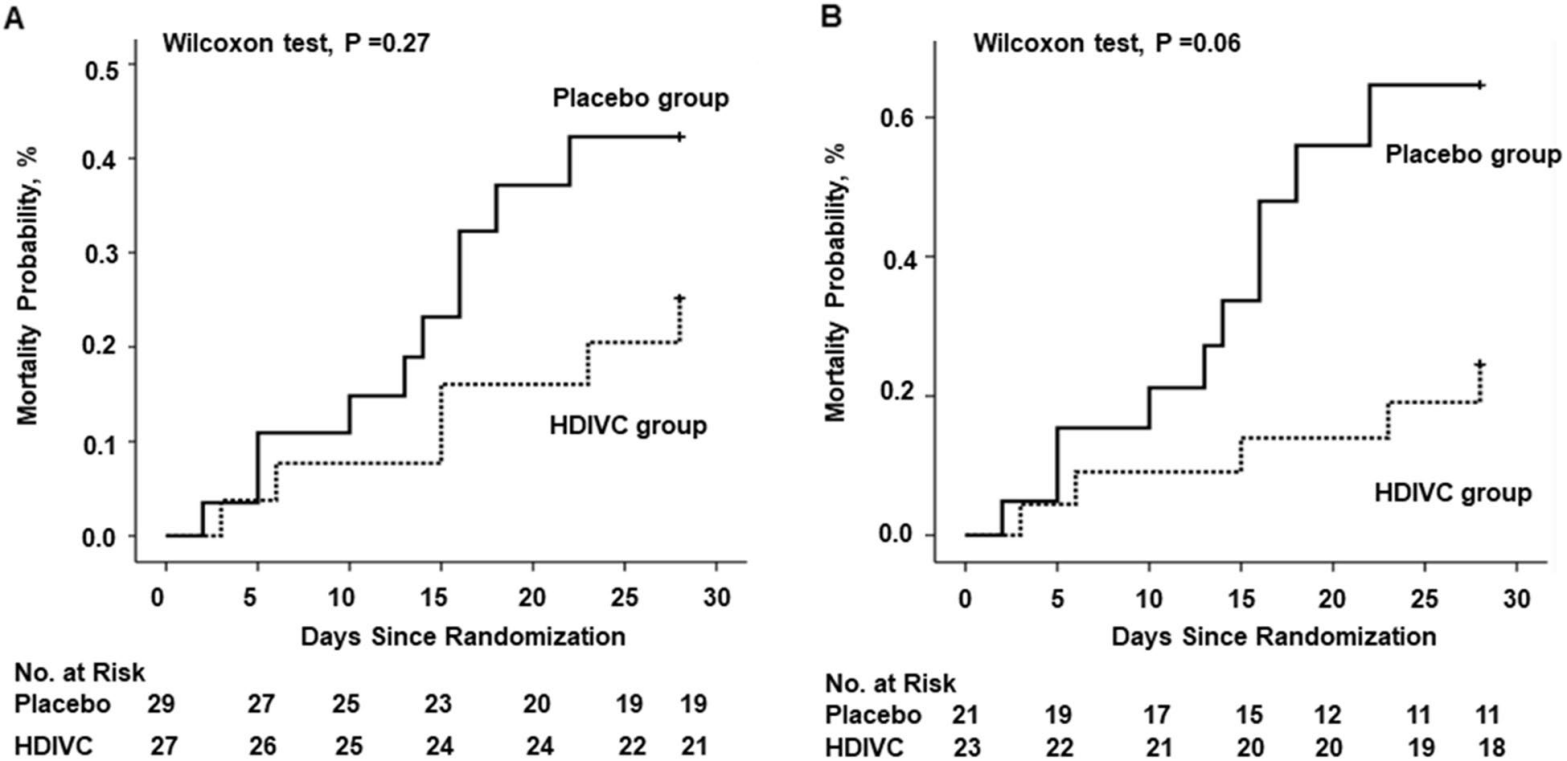
The 28-day mortality from randomization (day 1) to day 28. **a** Kaplan–Meier analysis was used to estimate the 28-day mortality, and survival curves were compared with the Wilcoxon test (*P* = 0.27) among patients with COVID-19. Cox regression was used for multiple comparisons (*P* = 0.31, HR, 0.50 [95% CI 0.2 to 1.8]). **b** Kaplan–Meier analysis was used to estimate the 28-day mortality and survival curves were compared with the Wilcoxon test (*P* = 0.06) among severe COVID-19 patients (baseline SOFA score ≥ 3). Cox regression was used as multiple comparisons (*P* = 0.07, HR, 0.32 [95% CI 0.10–1.06]). *HDIVC* high-dose intravenous vitamin C, *COVID-19* coronavirus disease 2019, *SOFA* Sequential Organ Failure Assessment

Kaplan–Meier analysis was used to estimate the 28-day mortality, and survival curves were compared with the Wilcoxon test (*P* = 0.27) among all the enrolled patients with COVID-19. Meanwhile, the Cox regression was used for comparisons (*P* = 0.31, HR, 0.50 [95% CI 0.2 to 1.8]). HDIVC infusion exhibited a trend of reduction in 28-day mortality (*P* = 0.06) in more severe patients (SOFA score ≥ 3) using univariate survival analysis, and Cox regression showed a similar results (*P* = 0.07, HR, 0.32 [95% CI 0.10–1.06]) (Fig. 3).

**Fig. 4:**
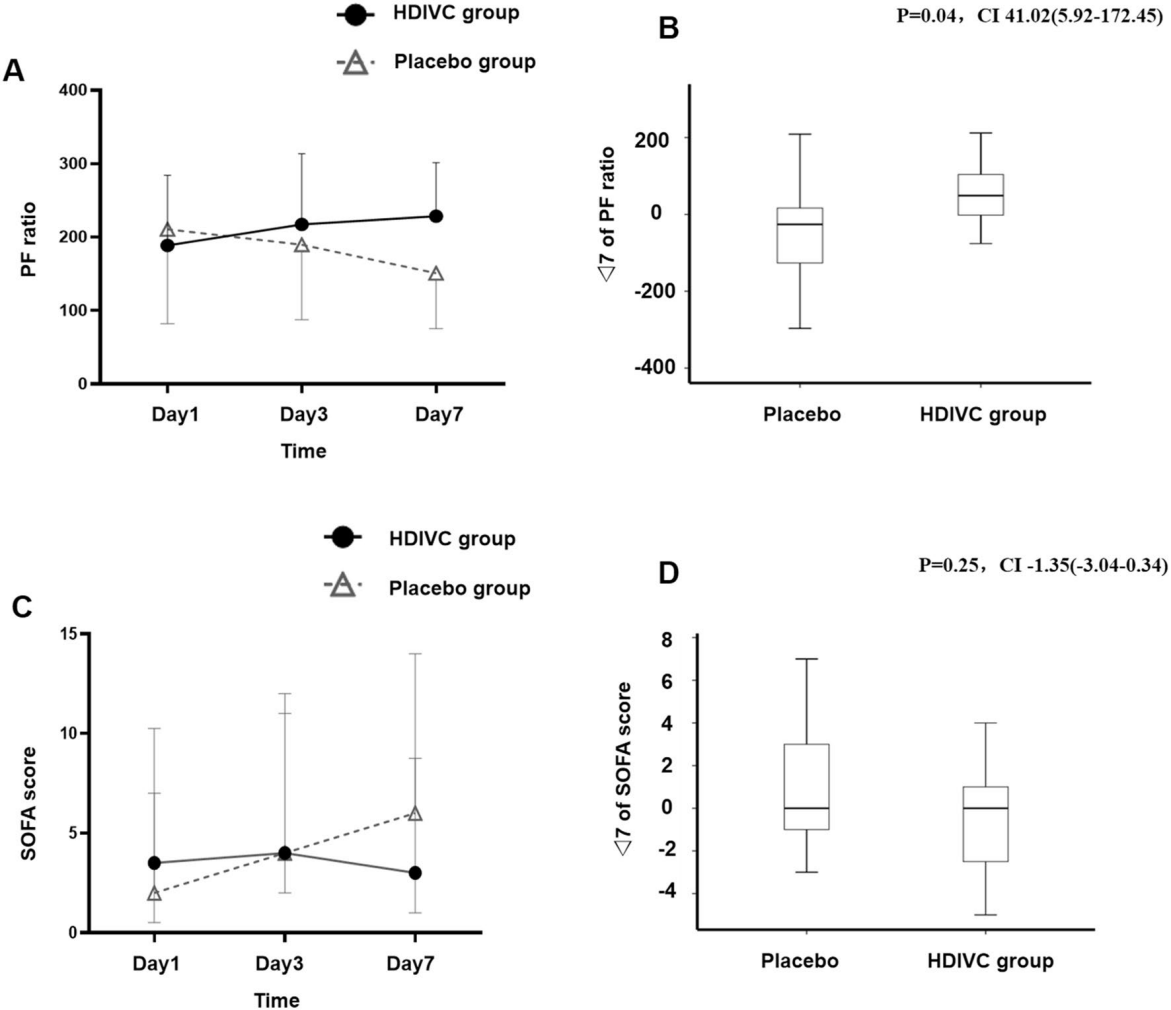
P/F and SOFA scores following high-dose intravenous vitamin C treatment. **a** The bars show the standard deviation (SD) of the mean. The P/F in both groups was approximately 200 at enrollment. After initiation of treatment, there was a steady rise in the P/F in the HDIVC group and a decline in the P/F in the placebo group (day 3: 217 vs. 189, 95% CI -34 to 90, *P* = 0.37; day 7: 229 vs. 151, 95% CI 33 to 122, *P* = 0.01). **b** △7 of P/F means the difference between the value from Day1 to Day7. Boxes represent the median and interquartile range (25th and 75th percentiles), and whiskers represent the range of values. The delta P/F ratio showed a different result in two groups (20.0 ± 96.68 vs. -51.88 ± 150.72, *P* = 0.04, 41.02 (5.92–172.45)). △7 was calculated by the difference between the value from Day 1 to Day 7. **c** The bars showed the interquartile range (IQR) of the median. There was no difference in the initial Sequential Organ Failure Assessment (SOFA) scores of the 2 groups at baseline (vitamin C vs placebo, median, 3.5[3.0–6.8] vs 2.0 [3.0–5.0]). After 7-day treatment, the median of SOFA score increased from 2.0 to 6.0 in the placebo group and slightly decreased from 3.5 to 3.0 in the HDIVC group, but there was no difference between the 2 groups. **d** △7 of SOFA scores means the difference between the value from Day1 to Day7. Boxes represent the median and interquartile range (25th and 75th percentiles), and whiskers represent the range of values. The delta SOFA scores showed no significant difference in two groups (0.0[-2.75-1.0] vs. 0.0[-1.0-3.5], P = 0.25, CI -1.35(-3.04-0.34)). △7 was calculated by the difference between the value from Day 1 to Day 7. *HDIVC* high-dose intravenous vitamin C, *SOFA* Sequential Organ Failure Assessment, P/F PaO_2_/FiO_2_*COVID-19* coronavirus disease 2019

As shown in Fig. 4, the median SOFA score increased from 2.0 to 6.0 in the placebo group while it slightly decreased from 3.5 to 3.0 in the HDIVC group on day 7. However, there was no statistically significant difference in SOFA scores between the two groups on days 3 and 7. During the 7-day treatment period, the P/F in the HDIVC group was 228.5 mmHg, and 150.7 mmHg in the control group (95% CI 33.2 to 122.5; *P* = 0.01), and improved over time in HDIVC group (Fig. 4). The delta P/F from day 1 to day 7 was (20.0 ± 96.7 in HDVIC and. -51.9 ± 150.7 in control, *P* = 0.04 (difference 41.0 (5.9–172.5)). IL-6 in the HDIVC group dropped to 9.4 pg/ml, while it increased to 158.0 pg/ml in the placebo group (95% CI -301.7, -29.8; P = 0.04) on day 7. There was no significant difference in other anticipated infectious indicators and inflammation biomarkers between the two groups (Table 3). In addition, total bilirubin was 8.40 in HDIVC group, and 14.9 in placebo group (95% CI -18.3 to -0.6; P = 0.03, Table 3). The ICU mortality of severe patients (baseline SOFA score ≥ 3, n = 42) was improved in the HDIVC group (P = 0.03, HR, 0.22 [95% CI 0.1–0.9]).

**Table 3:** Laboratory findings in a trial of HDIVC in patients with COVID-19

Variable	Day	Vitamin C (*n* = 27)	Placebo (*n* = 29)	Difference, coefficient (95% CI)	*P* Value
Leukocyte count, 10^9^	1	9.5 ± 5.0	11.6 ± 7.2	− 2.0(− 5.4 to -1.5)	0.26
	3	8.6[5.7–11.5]	8.4[7.1–12.2]	− 0.4(− 3.5 to 2.7)	0.67
	7	10.2 ± 6.7	9.6 ± 5.4	0.6(− 3.0 to 4.1)	0.74
Neutrophil count, 10^9^	1	8.2 ± 4.8	10.2 ± 7.1	− 2.0(− 5.3 to 1.4)	0.24
	3	6.2[4.5–10.5]	7.1[5.7–9.9]	− 0.5(− 3.5 to 2.5)	0.50
	7	8.1 ± 6.5	8.2 ± 5.5	− 0.1(− 3.6 to 3.4)	0.95
Neutrophil ratio, %	1	83.5 ± 9.6	85.8 ± 9.9	− 2.3(− 7.7 to -3.1)	0.39
	3	85.7[77.1–91.4]	83.3[75.5–91.8]	4.0(− 6.3 to 14.4)	0.70
	7	78.5 ± 15.8	81.7 ± 11.5	− 3.2(− 11.2 to 4.8)	0.42
IL-6	1	22.6[8.9–85.5]	54.7[12.3–145.5]	-6.2(-129.7 to 117.3)	0.61
	3	113.1[21.8–288.7]	37.2[5.6–85.3]	92.4(− 25.1 to 210.0)	0.07
	7	19.4[10.6–29.2]	158.0[15.3–259.6]	− 165.8(− 301.7 to − 29.8)	**0.04**
Lymphocyte count, 10^9^	1	0.6[0.4–1.0]	0.5[0.4–1.0]	0.1(− 0.2 to 0.4)	0.49
	3	0.6[0.3–1.0]	0.71[0.5–1.1]	− 2.6(− 8.6 to 3.4)	0.50
	7	0.8[0.4–1.1]	0.7[0.4–1.0]	1.1(− 0.8 to 3.0)	0.25
Lymphocyte ratio, %	1	9.7 ± 7.0	8.1 ± 7.3	1.6(− 2.3 to 5.6)	0.41
	3	10.1 ± 9.2	8.7 ± 4.9	1.4(− 2.7 to 5.4)	0.88
	7	13.1 ± 11.3	6.8[5.1–13.4]	3.3(− 2.1 to 8.8)	0.23
PCT, ng/mL	1	0.2[0.1–0.6]	0.2[0.1–0.5]	− 9.9(− 29.3 to 9.4)	0.80
	3	0.4[0.1–3.2]	0.3[0.1–1.1]	− 6.6(− 20.5 to 7.3)	0.84
	7	0.3[0.1–14.8]	0.2[0.1–0.7]	13.3(− 17.9 to 44.5)	0.18
CRP, mg/L	1	39.9[3.9–86.9]	56.8[40.2–100.2]	− 23.2(− 69.5 to 23.1)	0.19
	3	43.5[3.4- 65.7]	66.3[29.8–107.4]	− 4.8(− 68.1 to 58.5)	0.28
	7	29.5[11.0–110.9]	30.2[2.3–131.7]	− 12.6(− 75.3, 50.1)	0.68
Total bilirubin, umol/L	1	8.6[6.8- 15.6]	10.8[7.4–18.3]	− 1.5(− 7.3 to 4.4)	0.28
	3	8.4[6.7–16.1]	14.9[9.9–25.5]	− 9.7(− 18.3 to − 0.6)	**0.03**
	7	8.3[6.5–16.2]	15.3[9.0–27.7]	− 4.2(− 15.9 to 7.5)	0.11
Creatinine, umol/L	1	64.2[46.9–85.5]	64.2[52.0 -81.7]	26.4(− 50.9 to 103.7)	0.57
	3	60.3[37.7–80.4]	70.35[49.80–100.9]	2.5(− 39.9 to − 44.9)	0.15
	7	57.5[40.0–7]	63.50[51.7–104.5]	− 12.4(− 45.6 to 20.7)	0.13
BUN, mmol/L	1	7.11[4.48–11.10]	6.50[4.9–9.9]	9.3(− 8.8 to 27.4)	0.84
	3	7.6 ± 5.0	8.6[5.1–11.4]	− 2.1(-5.2 to − 1.0)	0.11
	7	8.5 ± 5.7	7.8[5.1–10.5]	− 0.7(− 4.1 to 2.7)	0.48
PT, s	1	13.3[12.4–14.6]	12.9[12.5–13.8]	− 0.6(− 2.4 to 1.2)	0.97
	3	13.9 ± 3.2	13.3[12.7–15.1]	− 0.29(− 2.0 to 1.4)	0.33
	7	13.0 ± 2.6	13.1[12.4–14.6]	− 0.3(− 1.7 to 1.1)	0.08

Data were expressed as mean ± standard deviation, as median [interquartile range]. Odds ratio with 95% CI were calculated by binary logistic regression for the rest. P values were calculated by logistic regression

*SD* standard deviation, *IQR* interquartile range, *HR* hazard ratio, *OR* odds ratio, *CI* confidence interval, *HDIVC* high-dose intravenous vitamin C, *COVID-19* coronavirus disease 2019, *SD* standard deviation; *IQR* interquartile range, *PCT* procalcitonin, *CRP* C-reactive protein, *BUN* blood urea nitrogen, *PT* prothrombin time, *IL-6* interleukin-6

### The differences of other treatments

Table 1 demonstrates the differences in other treatments between the two groups. There were no significant differences in corticosteroids, antiviral agents or antibiotics.

### Adverse events

During the 7-day infusion period, serum creatinine was 64.20[46.58–85.45] on day 1 and 57.50[39.95–71] umol/L on day 7 in HDIVC, versus 64.20[52.00 -81.70] on day 1 and 63.50[51.70–104.50] umol/L on day 7 in control group. Similarly, there were no changes in total bilirubin from day 1 to day 7 in HDIVC, while there was a slight increase from day 1 to day 7 in placebo. No other study-related adverse events were found, and no patients hadn’t finished the study due to SAEs.

## Discussion

This pilot trial shows that the addition of high-dose (24 g per day for 7 days) intravenous vitamin C to the standard-of-care treatment for severe COVID-19 did not affect ventilation-free days, but may provide a potential signal of benefit in oxygenation and IL-6. To our understanding, it was the first trial on a high dose of vitamin C infusion in patients with severe COVID-19.

Other previous studies suggested a protective role of vitamin C infusion in acute lung injury (ALI) and ARDS [21]. Moreover, the latest meta-analysis from eight vitamin C trials of a total of 685 patients indicated that vitamin C shortened the duration of mechanical ventilation in critically ill patients [27]. SARS-CoV-2 primarily affects the lung and causes pneumonia. Respiratory failure from ARDS is the leading cause of mortality from COVID-19 [32]. Similar to sepsis-induced ALI/ARDS, the rapid increase in cytokines in COVID-19 causes neutrophil sequestration in the lung, which damages the alveolar capillaries [9, 10]. In sepsis modeling of mice, parenterally infused VC demonstrated a protective effect on the lung [33, 34]. The potential mechanisms included limiting cytokine surges, improving alveolar fluid clearance, preventing vascular injury, restoring endothelial and alveolar epithelial integrity, and augmenting lung barrier cell function. In our study, the primary endpoint, mechanic ventilation-free days, was not demonstrated statistical significance due to the limited sample size, and late initiating HDIVC. However, the P/F increased, which was likely the result of pulmonary ventilation function improvement, based on the above mechanisms.

Previous clinical trials showed that HDIVC may reduce the extent of multiple organ failure and may improve the short-term outcomes of sepsis [19, 21], even though results in sepsis have been quite variable (ref Australian study/VICTAS just presented at ESICM). Additionally, plasma ascorbic acid levels were inversely correlated with the incidence of multiple organ failure and the risk of mortality [35]. We suspected that patients with worse organ dysfunction may have a more severe vitamin C deficiency, while high-dose intravenous VC effectively improved the deficiency and subsequently improved organ function [16]. Thus, the benefit was more significant in more severe COVID-19 patients with a higher baseline SOFA score in our study.

In this study, we chose 24 g of vitamin C infusion for 7 days. The main reason was based on two aspects: the efficacy and safety. The metabolism of vitamin C (VC) in the blood is very fast, only large dose and long course of VC supplement can maintain an adequate concentration in blood. In a previous study [19], 50 or 200 mg/kg/day (equivalent to 12 g/day) in 4 days VC treatment showed a signal of benefit in sepsis or ARDS patients. Similar daily doses were used in the Fowler paper (JAMA), which was associated with an improved outcome Thus, we tried to improve the efficacy by increasing the dosage and course in this trial. Actually, the 24 g dose is far less than the conventional IVC dose for cancer patients. In addition, high-dose VC has been clinically used for several decades and a recent NIH expert panel document states clearly that this regimen (1.5 g/kg body weight) is safe and without major adverse events (https://www.cancer.gov/about-cancer/treatment/cam/hp/vitamin-c-pdq). Therefore, we believe that this 24 g/day for 7 days is safe and more effective.

In addition, high levels of IL-6 were observed in patients with COVID-19 and might serve as a predictive biomarker for disease severity [5, 36, 37]. Mechanistically, IL-6 acts as a critical cytokine in the systemic inflammatory response [38], leading to a myriad of biological effects that contribute to pulmonary infiltration and organ damage [39, 40]. In a recent trial, tocilizumab [41], a recombinant humanized anti-human IL-6 receptor antibody, improved clinical symptoms by attenuating inflammation in COVID-19. The findings of the decline in IL-6 in our cohort were consistent with basic research showing that vitamin C inhibited the production and release of proinflammatory cytokines from human monocytes (IL-1, IL-2, IL-6, and TNF-α) [42]. Previous animal studies on SARS-CoV also demonstrated that inhibiting NF-κB, together with reduced IL-6 levels, could increase the survival rate in infected animals [37].

This study has several limitations. First, the study was started in the second half of the outbreak in China, and the number of qualifying COVID-19 patients decreased with the control of the epidemic so that we had to stop our trial before reaching the predefined sample size. Secondly, the initiation of vitamin C occurred more than 10 days after the first symptom, which may affect the efficacy of HDIVC. However, SARS-CoV-2 infection was characterized by mild symptoms initially, followed one week later by a rapid deterioration leading to hospitalization, and ARDS always occurred at the day 8 after the first symptom [4]. As in other randomized trial, administration of vitamin C was initiated shortly after the onset of ARDS [21], which started a couple of days earlier than our trial. Third, the absence of data on the monitoring of serum ascorbic acid concentration and assessment of viral load made it unclear whether vitamin C has direct antiviral activity against SARS-CoV-2. Fourth, we did not measure the anti-oxidative variables due to the complexity of the blood sample treatment, which was also an important feature for vitamin C. Finally, the imbalance in the patient gender distribution between the groups at baseline may have slightly influenced the outcomes.

## Conclusion

In summary, this pilot trial showed that HDIVC did not improve the primary endpoint, IMVFD28, but demonstrated a potential signal of benefit for critically ill COVID-19, with an improvement in P/F ratio. Nevertheless, further large-scale RCTs are still needed to confirm our understanding of the effect of HDIVC therapy in critically ill patients with COVID-19.

## Data Availability

The datasets used and analyzed during the current study are available from the corresponding author on reasonable request.
